# Multiview Clustering of Adaptive Sparse Representation Based on Coupled P Systems

**DOI:** 10.3390/e24040568

**Published:** 2022-04-18

**Authors:** Xiaoling Zhang, Xiyu Liu

**Affiliations:** Academy of Management Science, Business School, Shandong Normal University, Jinan 250014, China; 2020020963@stu.sdnu.edu.cn

**Keywords:** multiview clustering (MVC), manifold learning, sparse representation, P system

## Abstract

A multiview clustering (MVC) has been a significant technique to dispose data mining issues. Most of the existing studies on this topic adopt a fixed number of neighbors when constructing the similarity matrix of each view, like single-view clustering. However, this may reduce the clustering effect due to the diversity of multiview data sources. Moreover, most MVC utilizes iterative optimization to obtain clustering results, which consumes a significant amount of time. Therefore, this paper proposes a multiview clustering of adaptive sparse representation based on coupled P system (MVCS-CP) without iteration. The whole algorithm flow runs in the coupled P system. Firstly, the natural neighbor search algorithm without parameters automatically determines the number of neighbors of each view. In turn, manifold learning and sparse representation are employed to construct the similarity matrix, which preserves the internal geometry of the views. Next, a soft thresholding operator is introduced to form the unified graph to gain the clustering results. The experimental results on nine real datasets indicate that the MVCS-CP outperforms other state-of-the-art comparison algorithms.

## 1. Introduction

At present, many fields have accumulated a large amount of data from which cluster analysis can mine useful knowledge. Cluster analysis has been effectively applied to text mining [1], information retrieval [2], pattern recognition [3], molecular biology [4], etc. With the rapid growth of multimedia technology and the widespread deployment of the Internet of Things, multiview data has become more common and public [5]. Due to the limitations of traditional clustering algorithms, multiview clustering has become a research hotspot. Multiview clustering essentially utilizes the input of multiple characteristic views of the data. It merges these feature views to acquire an optimized model, which is more efficient than traditional single-view clustering. Then, the instance is divided into different clusters [6,7]. There are two important principles of the multiview clustering algorithm, namely, the principle of complementarity and consensus. The former can describe instances more comprehensively, and multiple views can complement each other. The latter is designed to maximize consistency between multiple different views.

In recent years, multiview clustering has developed rapidly. In theory, most existing multiview clustering methods can be divided into four categories: graph-based techniques, non-negative matrix factorization, multikernel clustering, and deep multiview clustering. The multiview spectral clustering method and the subspace clustering method are graph-based multiview clustering methods. The former commonly applies spectral embedding [8], tensor learning [9,10] and relaxation matrix methods [11]. The latter is more effective in processing high-dimensional data. Methods such as sparse representation and low-rank representation are usually adopted to gain subspace self-representation [12,13,14,15]. The non-negative matrix factorization method adopts multiple normalizations, double regularization, and graph regularization strategies of non-negative matrix factorization factors to improve the performance of multiview clustering [16,17,18]. Multikernel clustering fuses linear kernels, polynomial kernels, Gaussian kernels and other kernel functions as base kernels. Meanwhile, it combines kernel norm minimization [19], tensor kernel norm [20], non-convex norm [21], extreme learning machine [22] and other methods to realize the clustering process.

Deep multiview clustering is another development direction of current multiview clustering. Constructing category-level correspondences of unaligned data with the latent space learned by neural networks in partial-view-aligned clustering [23,24]. Robust multiview clustering with incomplete information addresses the partially view-unaligned problem and the partially sample-missing problem under a unified framework combined with neural networks [25]. Moreover, the concepts of intelligent algorithms such as particle swarm optimization (PSO) [26,27,28], the Boltzmann machine [29], encoder [30], and convolutional neural network (CNN) [31] are introduced into multiview clustering to solve practical application problems. Since the graph-based clustering methods have advantages in terms of accuracy, this paper will focus on them.

In multiview clustering, the construction of a single-view similarity matrix and the formation of a unified graph matrix are important issues. Zhan et al. [32] put forward an unsupervised multi-feature learning method based on graph construction and dimensionality reduction (MVGL) to maximize the use of the correlation between different features. The weights of the affinity matrix are learned through well-designed optimization problems rather than fixed functions. In addition, a rank constraint is imposed on the Laplacian matrix of the global graph to achieve an ideal neighbor assignment. Maria and Ivica [12] learned the joint subspace representation by constructing an affinity matrix shared by all views while encouraging the sparsity and low rank of the solution. The proposed multiview low-rank sparse subspace clustering (MLRSSC) algorithm strengthens the consistency between affinity matrices of the pairs of views. Wang et al. [33] presented a graph-based system for multiview clustering.

The working principle of GBS is to employ the nearest neighbor method to effectively construct the similarity matrix after extracting the data feature matrix of each view. Then, an iterative optimization algorithm is adopted to automatically weight each similarity matrix to learn a unified affinity matrix. It can directly gain the final cluster labels in the unified graph. Peng et al. [34] set the neighbor size to 10 and used the cosine distance as a metric to construct a similarity matrix, and then updated it iteratively with an objective function that includes geometric consistency. GMC [35] automatically weights and merges the similarity matrix of each view to generate a unified graph matrix. The two improve each other by means of iterative optimization algorithms and give the final cluster directly without additional algorithms. Tan et al. [7] proposed a two-step multiview clustering method that exploits sparse representation and adaptive graph learning to optimize the similarity matrix of a single view, and retains the internal structural characteristics of each view. Further, the global optimal matrix is obtained through adaptive weighted cooperative learning for each view. Huang et al. [36] merged the consistency and diversity of multiple views into a unified framework to form a “consistent and divergent multiview graph” (CDMGC). At the same time, an alternating iterative algorithm combines the consistency part with the automatically learned weights, and the consistency part is further integrated into the target affinity matrix. Finally, the clustering label of each instance is directly assigned.

Membrane computing (also known as a P system) is a distributed parallel computing model that Professor Păun proposed in 1998 inspired by the structure and function of biological cells [37]. Since it was put forward, its calculation model has been proved to have the computing power equivalent to the Turing machine [38]. The neural P system is the third-generation neural membrane computing model inspired by discrete neurons whose information is encoded by neurons’ spike number and spike time. In addition, there are currently cell-like P systems and tissue-like P systems [39]. The development of membrane computing (P system) is mainly in theoretical research and application research. In theoretical research, the research on the parallel computing capabilities of various P systems and solving NP problems are flooding in [40,41,42,43]. In terms of application research, membrane computing has been widely used in spectral clustering [44,45] and density peak clustering [46]. 

Most of the above algorithms adopt the concept of neighbors when initially constructing the similarity matrix, but most of them used a fixed number of neighbors manually determined. However, multiview data is usually collected from different measurement methods, such as images, videos, etc. The noise, damage, and even view-specific attributes of different data sources will be different, so the number of neighbors should be different when the similarity matrix of each view is constructed. Simultaneously, most of the existing multiview clustering algorithms utilize iterative optimization algorithms when merging into a unified graph matrix and decomposing it into subproblems for disposing of. Although higher accuracy can be achieved, the calculation time is increased. Therefore, regarding the issue above, this paper proposes a multiview clustering of adaptive sparse representation based on a coupled P system (MVCS-CP) and verifies the clustering performance. The main contributions of this paper are as follows:

(1) A new coupled P system is proposed, which integrates the construction of a single view matrix and the formation of a unified graph into the P system to perform clustering tasks.
(2) To construct the similarity matrix of each view, this paper introduces a natural neighbor search algorithm without parameters, which can automatically determine the number of neighbors in each view. After that, sparse representation and various learning methods are imported to construct the similarity matrix to preserve the internal geometry of the views.
(3) In forming a unified graph, this paper adopts a soft thresholding operator to learn a consistent sparse structure affinity matrix from the similarity matrix of each view and then obtain the clustering result. Iterative optimization is not required, and better clustering results can be captured and obtained quickly.
(4) Nine multiview data sets are employed to simulate and verify the clustering performances of MVCS-CP.

The remaining parts of this paper are arranged as follows: Section 2 introduces the related concepts of the P system and graph learning and other related works. The proposed multiview clustering of adaptive sparse representation based on a coupled P system (MVCS-CP) is outlined in Section 3. Section 4 details the experimental results and analysis the performance of the algorithm. The summary of this paper and the perspective for future work are given in Section 5.

## 2. Related Work

### 2.1. Notations

In this paper, vectors, matrices, and scalars are represented by bold lowercase letters (x), bold uppercase letters (X), and lowercase letters (x), respectively. $X=\left\{X^{1},X^{2},\cdots ,X^{m}\right\}$ denotes a dataset with m views, where $X^{v}\in R^{d^{v}\times n}$. Its jth column vector is represented as $x_{j}$, and the (i,j) instance is $x_{ij}$. I represents the identity matrix and 1 represents a column vector with entries as one. Tr(X) and ${\left||X|\right|}_{F}$ is the trace and Fresenius norm of X. For a vector x, its $\ell_{p}$ norm is ${\left||x|\right|}_{p}$. L denotes the Laplacian matrix constructed by the similar matrix $S^{n\times n}$.

### 2.2. Graph-Based Clustering and Graph Learning

Supposing that all elements in the similarity matrix $S^{n\times n}$ are non-negative, the relevant properties of the Laplacian matrix L can be obtained [47,48].

**Theorem** **1. ** *The multiplicity c of the eigenvalue 0 of the Laplacian matrix **L** is equal to the number of connected components of the similarity matrix **S***.

That is, when the constraint condition of rank (L)=n−c is fulfilled, then the similarity matrix S is the most suitable neighbor allocation and the data points have been divided into c clusters [49]. If the sum of the first c smallest eigenvalues of the Laplacian matrix L is equal to 0 and satisfies the constraint of rank (L)=n−c, $\sum_{i=1}^{c}\lambda_{i}=0$, where $\lambda_{i}$ refers to the i-th smallest eigenvalue of L. Hence, according to Fan’s theorem [50], it has:

(1) $$\begin{array}{c} \overset{c}{\underset{i=1}{\sum }}\lambda_{i}=\underset{F}{\min}tr\left(F^{T}LF\right)\\ s.t.F\in R^{n\times n},\;F^{T}LF=I \end{array}$$

where $F^{T}=\left(f_{1},f_{2},\ldots ,f_{n}\right)$ is the eigenvector matrix of $L=D-\left[\left(S^{T}+S\right)/2\right]$, $D=\sum_{i=1}^{m}{\left(S^{T}+S\right)}_{ii}/2$ is a diagonal matrix.

### 2.3. Natural Neighbours

On the basis of previous studies, Zhu et al. [51,52] systematically summarized and defined the concept of natural neighbors. For data objects in $X^{v}\in R^{d^{v}\times n}$, its natural neighborhood stable structure can be expressed as $\left(\forall x_{i}\right)\left(\exists x_{j}\right)\left(k\in N\right)\wedge \left(i\neq j\right)\to \left(x_{i}\in NN_{k}\left(x_{j}\right)\right)\wedge (x_{j}\in NN_{k}\left(x_{i}\right)$, where $NN_{k}\left(x_{i}\right)=\{x_{j}\in X|d\left(x_{i},x_{j}\right)\leq d\left(x_{i},kn\right)\}$ is the kth nearest neighbor of $x_{i}$, $d\left(x_{i},kn\right)$ is the distance of the *k*th nearest neighbor of $x_{i}$.

**Definition** **1.**  **(*The Natural Characteristic Value Ncv*)***Ncv is equivalent to the number of natural neighbors (That is, k) of the data point*x.

(2) $$Ncv=\min\{k|\overset{n}{\underset{i=1}{\sum }}f\left(Nb_{k}\left(x_{i}\right)\right)=0\;or\;\overset{n}{\underset{i=1}{\sum }}f\left(Nb_{k}\left(x_{i}\right)\right)=\overset{n}{\underset{i=1}{\sum }}f\left(Nb_{k-1}\left(x_{i}\right)\right)\}$$

where $Nb_{k}\left(x_{i}\right)$ is the number of reverse neighbors ($RNN\left(x_{i}\right)=\{x\in X|x_{i}\in NN_{k}\left(x\right)\}$) of $x_{i}$ in the kth iteration. Furthermore, $f\left(x\right)=\{\begin{array}{c} 0,\;otherwise\\ 1,\;if\;x==0 \end{array}$.

**Definition** **2.**  **(** ***The Natural Neighbors*** **)** *The natural neighbors of the object* x *in the data set are the **k** nearest neighbors, expressed as NaN (* x *).*

### 2.4. P System

**Figure 1:**
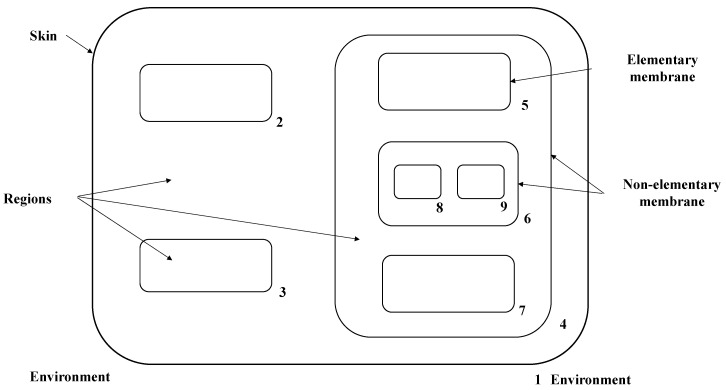
The basic membrane structure of the cell-like P system.

The cell-like P system is the first generation of the membrane computing model. The structure is shown in Figure 1. It is divided into basic membranes (such as 2, 3, 5, 7, 8 and 9) and non-basic membranes (such as 1, 4 and 6). Membrane 1 is also called the skin membrane, isolating the P system from the external environment.

**Figure 2:**
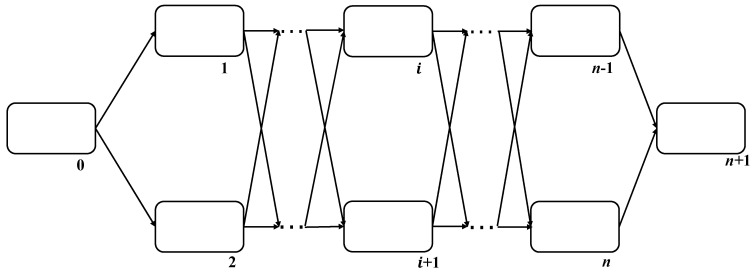
The basic membrane structure of the tissue-like P system.

The tissue-like P system is composed of multiple single membrane cells, which rely on designated channels for communication. The basic membrane structure of the tissue-like P system is shown in Figure 2. The initial object is in the input cell (membrane 0), using rules and communication mechanisms to correspond between cell 1 and cell *n*. Cell *n* + 1 is the output cell stored in the obtained results.

## 3. Multi-View Clustering of Adaptive Sparse Representation Based on Coupled P Systems

**Figure 3:**
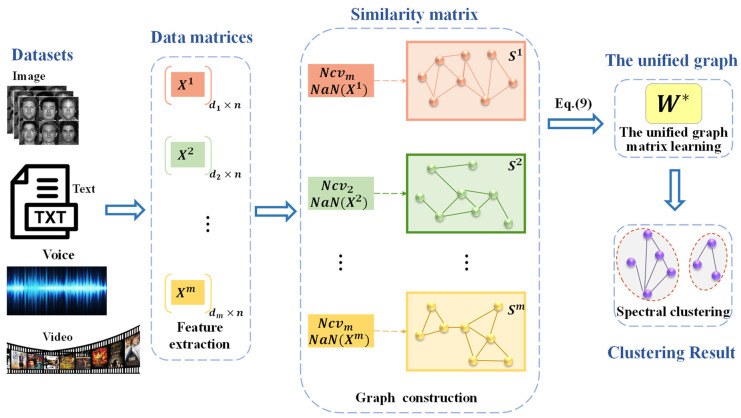
The flow chart of the MVCS-CP algorithm.

This section puts forward the multiview clustering method of adaptive sparse graph learning based on a coupled P system. At first, we elaborated on the general framework of the defined coupled P system. After that, different evolution rules and manipulation, including the construction of a similarity matrix of each view after the number of neighbors is determined, the formation of the unified graph, and clustering are discussed in turn. In addition, it explains the communication rules between different subsystems if there is a synapse between cells. The flow chart of the MVCS-CP algorithm is shown in Figure 3.

### 3.1. The General Framework of the Proposed Coupled P System

**Figure 4:**
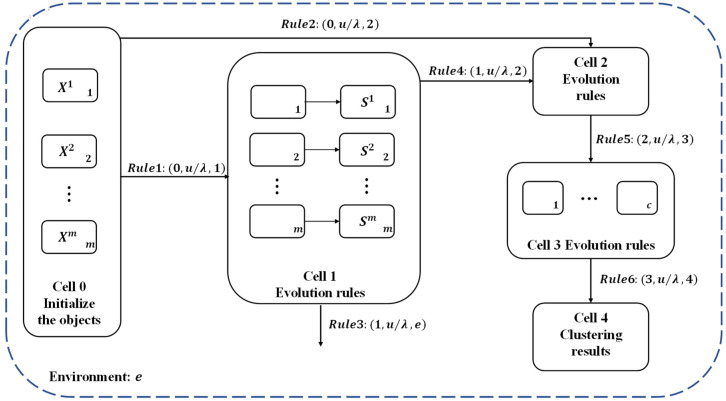
The basic structure of the coupled P system.

The proposed coupled P system (MVCS-CP) is formed based on the tissue P system by adding the relevant knowledge of the cell P system. As shown in Figure 4, it is the basic structure of the coupled P system (MVCS-CP), showing part of the basic information in the algorithm system.

**Definition** **3. ** *The formal definition of the MVCS-CP system is*

$$\prod =\left(\Gamma ,\varepsilon ,syn,\sigma_{0},\cdots ,\sigma_{t},R,in,out\right)$$

*where*

- $\Gamma =\left\{X^{1},X^{2},\ldots ,X^{m},S^{1},S^{2},\ldots ,S^{m},NaN\left(x\right),Ncv,Nb\left(x\right),W,D,L,para,c,\right\}$. $X^{i}$, $S^{i}$*represent the original data of*m*views and the similarity matrix corresponding to each view, respectively.*NaN(x)*is the natural neighbor of the data point*x*in the view.*Ncv*refers to the characteristic natural value, and the number of reverse neighbors of*x*is denoted as*Nb(x). W*represents the learned uniform unified graph matrix.*D*and*L*indicate the degree matrix and Laplacian matrix, respectively. The parameters*para*and*c*respectively refer to the parameters required for the experiment and the number of clusters.*
-
$\varepsilon =\left\{X^{1},X^{2},\ldots ,X^{m},para,c\right\}\subseteq \Gamma$ *is the initial objects in the coupled systems.*
-
syn={{0,1},{0,3},{1,2},{2,3},{3,4}} *signifies the synapse between cells, whose main function is to connect cells and make them communicate with each other.*
-
$\sigma_{0},\cdots ,\sigma_{t}$ *denotes the cells (membrane) in the system.* t *depends on the number of views and the number of clusters in the data set, that is, the total number of cells in the system.*
-
R *represents a collection of communication rules and evolution rules in the system. The role of evolution rules is to modify objects and communication rules are used to transfer objects between cells (membranes).*
-
in *is cell 0, which is the input membrane.* out *is cell 5, output membrane, used to store the final clustering results.*

### 3.2. The Evolution Rules

The input cell 0 initializes the data object. It transmits the data and corresponding parameters of the multiview to cell 1 to determine the Natural Characteristic Value and construct the similarity matrix of each view. At the same time, the number of clusters c is transported from cell 0 to cell 2 to form a new cluster sample for *k*-means. The rule R0 can be described in detail as:

- $R_{01}=\left\{X^{1},X^{2},\ldots ,X^{m},para\to X^{1},X^{2},\ldots {,}_{go{\left[\right]}_{1}}\right\}$
- $R_{02}=\left\{c,para\to c,para{,}_{go{\left[\right]}_{2}}\right\}$

The output cell 4 stores the clustering results obtained by the algorithm. $R_{4}\neq \emptyset$.

#### 3.2.1. The Evolution Rules of Determining *Ncv* and Constructing Similarity Matrix in Cell 1

In practice, when constructing the similarity matrix, it prefers data objects having similarities with neighbors. Then the choice of the number of neighbors is an important influencing factor. Most of the traditional algorithms are manual input obtained from experience, such as 10, 15. However, the source channel of each view in the multiview data is different, and the number of neighbors should be different. Therefore, in order to promote the accuracy of the algorithm, a non-parameter natural neighbor search algorithm is adopted in this paper to automatically determine the number of neighbors in each view.

In summary, the detailed evolution rules for determining natural characteristics in cell 1 are shown in rule *R*_1_:

- *R*_11_ (Iterative search rules): At the kth iteration, for each data point $x_{i}$ in the single view $X^{v}$, we search for its rth neighbor $x_{j}$ using a KD tree. After that, $Nb\left(x_{j}\right)=Nb\left(x_{i}\right)+1,NaN_{k}\left(x_{i}\right)=NaN_{k-1}\left(x_{i}\right)\cup x_{j}$ correspond to the concepts in Section 2.3. NaN(x) will be transported to the related subcell to construct the similarity matrix $S^{\nu }$.
- *R*_12_ (Iterative stop rules): If the number of reverse neighbors Nb(x) of data point x does not change or Nb(x)==0, the evolution rules stop.
- *R*_13_ (Determine the Ncv rule): The natural characteristic value Ncv is calculated by Equation (2), which is equivalent to the number of neighbors k, and then k is transmitted to the relevant subunits to prepare for the construction of $S^{\nu }$.

Manifold learning is finding a low-dimensional manifold in a high-dimensional space and exploring the corresponding embedding mapping to achieve dimensionality reduction or data visualization [53,54]. The general explanation is that if two data objects are close, they are also close in the embedding graph. Noise and outliers have always been factors that affect the final clustering results. Research [55] has found that sparse representation is robust to them. Therefore, this paper introduces manifold learning and sparse representation to construct the similarity matrix. In detail, the similarity matrix $S^{v}$ of each view $X^{v}$ is obtained by solving the following problems:

(3) $$\begin{array}{c} \underset{S^{v}}{\min}\overset{n}{\underset{i,j=1}{\sum }}||x_{i}^{v}-x_{j}^{v}||{}_{2}^{2}s_{ij}^{v}+\alpha \overset{n}{\underset{i}{\sum }}||s_{i}^{v}||{}_{1}\\ s.t.s_{ii}^{v}=0,s_{ij}^{v}\geq 0. \end{array}$$

When $s_{i}^{v}$ is normalized with $1^{T}s_{i}^{v}=1$, the second term of Equation (3) becomes a constant. Namely, the normalization and the sparse representation on $s_{i}^{v}$ are equivalent. Then, problem (3) can change into:

(4) $$\begin{array}{c} \underset{S^{v}}{\min}\overset{n}{\underset{i,j=1}{\sum }}||x_{i}^{v}-x_{j}^{v}||{}_{2}^{2}s_{ij}^{v}\\ s.t.s_{ii}^{v}=0,s_{ij}^{v}\geq 0,1^{T}s_{i}^{v}=1. \end{array}$$

Suppose that problem (4) has a trivial solution. The value of the only data point with the smallest distance to $x_{i}^{v}$ is 1, while the value of all other data points is 0. Now, adding a before question (2), its expression is

(5) $$\;\begin{array}{c} \underset{S^{v}}{\min}\overset{n}{\underset{i,j=1}{\sum }}||x_{i}^{v}-x_{j}^{v}||{}_{2}^{2}s_{ij}^{v}+\beta \overset{n}{\underset{i}{\sum }}||s_{i}^{v}||{}_{2}^{2}\\ s.t.s_{ii}^{v}=0,s_{ij}^{v}\geq 0,1^{T}s_{i}^{v}=1. \end{array}$$

If we only pay attention to the second item of Equation (5), the prior can be regarded as the similarity value of each data point to $x_{i}^{v}$, that is, 1/n. As can be seen from the above problem, Equation (5) is independent in terms of each data object i. Therefore, the following problems can be solved separately for each data object i:

(6) $$\;\begin{array}{c} \underset{s_{i}^{v}}{\min}\overset{n}{\underset{j=1}{\sum }}||x_{i}^{v}-x_{j}^{v}||{}_{2}^{2}s_{ij}^{v}+\beta \overset{n}{\underset{i}{\sum }}||s_{i}^{v}||{}_{2}^{2}\\ s.t.s_{ii}^{v}=0,s_{ij}^{v}\geq 0,1^{T}s_{i}^{v}=1. \end{array}$$

We adopt $d_{ij}$ to represent $||x_{i}^{v}-x_{j}^{v}||{}_{2}^{2}$ and $d_{i}$ is its vector. Afterward, it can depict problem (6) in vector form:

(7) $$\underset{s_{i}^{v}}{\min}||s_{i}^{v}+{\frac{d_{i}}{2\beta }||}_{2}^{2}$$

Problem (7) can be solved by a closed-form solution, as shown in [56]. As mentioned at the beginning of this section, it has been said that the construction of the similarity matrix requires the number of neighbors k. k has been gained in the front, which is equivalent to the natural characteristic value Ncv.

To sum up, the evolution rules for constructing the similarity matrix of each view in cell 1 are as follows:

- *R*_14_ (Lagrange function rule): The Lagrange function of Equation (17) is $ℒ\left(s_{i}^{v},\epsilon ,\zeta \right)=||s_{i}^{v}+{\frac{d_{i}}{2\beta }||}_{2}^{2}-\epsilon \left(1^{T}s_{i}^{v}-1\right)-\zeta^{T}s_{i}^{v}$
- *R*_15_ (Constraint rule): Based on the Karush–Kuhn–Tucker constraint, the optimal solution ${\hat{s}}_{ij}^{v}={\left(-\frac{d_{ij}}{2\alpha }+\epsilon \right)}_{+}$ can be acquired, where ${\left(a\right)}_{+}=max\left(a,\;0\right)$. As a result of the constraints $1^{T}s_{i}^{v}=1$, it has $\epsilon =\frac{1}{k}+\frac{1}{2k\beta }\overset{k}{\underset{j=1}{\sum }}h_{ij}$.
- *R*_16_ (Determining β rule): Since there are only k non-zero values in $s_{i}^{v}$, β has a maximum value, which is conveyed as $\beta =\frac{k}{2}d_{i,k+1}-\frac{k}{2}\Sigma j=1^{k}d_{ij}$.
- *R*_17_ (Getting the $s_{i}^{v}$ rule): The j-th element of $s_{i}^{v}$ is as follows:
  $$s_{ij}^{v}=\{\begin{array}{cc} \frac{d_{i,k-1}-d_{ij}}{kd_{i,k+1}-\sum_{h=1}^{k}b_{ih}} & j\leq k\\ 0 & j>k \end{array}.$$

Through the above evolution rules, the number of neighbors can be automatically determined. The introduced manifold learning and sparse representation are robust to noise and outliers, and thus the similarity matrix $S^{v}$ of each view is obtained in cell 1.

#### 3.2.2. The Evolution Rules of Constructing the Unified Graph Matrix, Degree Matrix, Laplacian Matrix in Cell 2

The unified graph matrix W is to merge each similarity matrix $S^{v}$ of multiple views into an affinity matrix to execute the subsequent clustering algorithm and obtain the final clustering result. Enlightened by previous models, this paper leads a soft thresholding operator into the unified graph affinity matrix based on the following two principles:

(1). The unified graph matrix W and the similarity matrix $S^{v}$ of each view tend to be as consistent as possible.
(2). The unified graph matrix W is sparse, which can further alleviate the noises generated by different views.

In order to construct the unified graph matrix as quickly as possible, the objective function is:

(8) $$\;\underset{W}{\min}\overset{m}{\underset{v=1}{\sum }}||S^{v}-{W||}_{F}^{2}+para{\left||W|\right|}_{0}$$

The first item of Equation (8) can satisfy the principle (1), while the second item satisfies the principle (2). Due to the $\ell_{0}$-norm minimization, the solution of Equation (8) is an NP-hard problem. According to previous studies on sparse learning [57,58], Equation (8) can be rewritten as:

(9) $$\;\underset{W}{\min}\overset{m}{\underset{v=1}{\sum }}||S^{v}-{W||}_{F}^{2}+para{\left||W|\right|}_{1}$$

where ${\left||W|\right|}_{1}$ is the convex relaxation of ${\left||W|\right|}_{0}$, and then Equation (9) can be expressed as:

(10) $$\begin{array}{cc} & \min_{W}||S^{1}-{W||}_{F}^{2}+||S^{2}-{W||}_{F}^{2}+,\ldots ,+||S^{m}-{W||}_{F}^{2}+para{\left||W|\right|}_{1}\\ \Rightarrow & \underset{W}{\min}\overset{m}{\underset{v=1}{\sum }}||S^{v}||{}_{F}^{2}-2Tr\left(\overset{m}{\underset{v=1}{\sum }}S^{v}W^{T}\right)+m{\left||W|\right|}_{F}^{2}+para{\left||W|\right|}_{1}\\ \Rightarrow & \underset{W}{\min}\frac{1}{m}\overset{m}{\underset{v=1}{\sum }}||S^{v}||{}_{F}^{2}-2Tr\left(\frac{1}{m}\overset{m}{\underset{v=1}{\sum }}S^{v}W^{T}\right)+{\left||W|\right|}_{F}^{2}+\frac{para}{m}{\left||W|\right|}_{1}\\ \Rightarrow & \underset{W}{\min}||\frac{1}{m}{\overset{m}{\underset{v=1}{\sum }}S^{v}||}_{F}^{2}-2Tr\left(\frac{1}{m}\overset{m}{\underset{v=1}{\sum }}S^{v}W^{T}\right)+{\left||W|\right|}_{F}^{2}+\frac{para}{m}{\left||W|\right|}_{1}+cons \end{array}$$

where cons is the constant item to be balanced, that is

(11) $$cons=\frac{1}{m}\overset{m}{\underset{v=1}{\sum }}||S^{v}||{}_{F}^{2}-||\frac{1}{m}{\overset{m}{\underset{v=1}{\sum }}S^{v}||}_{F}^{2}$$

All in all, the evolution rules for constructing the unified graph matrix in cell 2 are shown in rule *R*_2_:

- *R*_21_ (Removing the cons rule): Removing the cons, then the problem (11) is redefined as $\underset{W}{\min}||T-{W||}_{F}^{2}+\frac{para}{m}{\left||W|\right|}_{1}$, where $T=\Sigma_{v=1}^{m}S^{v}/m$.
- *R*_22_ (Soft-thresholding operator rule): Based on the above, when μ>0, the soft-thresholding operator is introduced here:
  $$S_{\mu }\left(x\right)=\{\begin{array}{cc} x-\mu , & x>\mu \\ x+\mu , & x<-\mu \\ 0, & otherwise \end{array}.$$
- *R*_23_ (Obtaining W rule): By conducting the $S_{\mu }$ element-wise, it can be extended to the matrix. In addition, as shown in [52], the approximate solution to problem (13) is $W^{*}=S_{\frac{para}{2m}}\left(T\right)$.
- *R*_24_ (Constructing the Degree Matrix rule): According to $D_{ii}=\sum_{j=1}^{n}W_{ij}$, the degree matrix D is gained.
- *R*_25_ (Constructing the Laplacian Matrix rule): In terms of the Laplacian Matrix, it is based upon W and D, $L=I-D^{-1/2}WD^{-1/2}$.

#### 3.2.3. The Evolution Rules of K-Means in Cell 3

On the basis of a unified graph matrix, this section adopts the spectral clustering method to acquire the final clustering results. The evolution rules are shown in *R*_3_.

- *R*_31_ (Building new cluster instances rule): The formation of clustering new instances is conducive to K-means clustering. We select the eigenvectors $U=\left\{u_{1},u_{2},\cdots u_{c}\right\},U\in R^{n*c}\;$ corresponding to the first c eigenvalues of L, and standardize it to obtain $Y_{ij}=U_{ij}/{\left(\sum_{j}U_{ij}^{2}\right)}^{1/2}$.
- *R*_32_ (Randomly selecting clustering centers rule): Among the n points of Y, it randomly selects c points as the initial clustering centers and stores them in the subcells.
- *R*_33_ (Clustering rule): After that, the distance from each instance to each cluster center is computed in the subcells simultaneously and transported to cell 3. Finally, the instances are allocated based on the principle of minimum distance to form c different clusters in cell 3.
- *R*_34_ (Outputting result rules): For clusters divided in accordance with rule *R*_35_, it takes the current average distance of each cluster as the new cluster center. Comparing the current cluster center with the previous cluster center, if there is a change, it repeats rule *R*_35_. Conversely, the result of clustering is outputted to cell 4.

### 3.3. The Communication Rules between Different Cells

In the MVCS system, communication between cells relies on the synapses between different cells. Membranes have distinct functions, such as initializing objects, executing algorithms, and outputting clustering results. In that way, the ordered communication between membranes makes the whole algorithm more efficient.

The rules of communication between different cells are as follows:

(1) Unidirectional transport between cells. u is a string containing the object. λ is the empty string.
  - Rule 1: (0,u/λ,1): It feeds u containing the original data X of m views into cell 2 for the determination of the similarity matrix for each view.
  - Rule 2: (0,u/λ,2): The u including the parameter para and the number of clusters c are transferred to cell 3 to format the unified graph matrix and construct the degree matrix and the Laplacian matrix.
  - Rule 4:(1,u/λ,2): The string u of similarity matrix S for each view produced by cell 1 is transported to cell 2 for the construction of the unified graph matrix.
  - Rule 5: (2,u/λ,3): It conveys the string u containing the Laplacian matrix and the number of clusters c to cell 3 for K-means clustering.
  - Rule 6: (2,u/λ,3): The string u of clustering results generated by K-means is transmitted to cell 4 for storage.
(2) Unidirectional transport between cells and the environment.
  - Rule 3:(0,u/λ,2): It transports the string u of the resulting reverse neighbor Nb(x) into the environment to release.

## 4. Experiments

In this section, we verify the performance of MVCS-CP on the real multiview dataset. All experiments were carried out in the MATLAB 2016a environment under the computer with Intel Core i7-2.9G CPU, 16 GB RAM, and Windows 10 64-bit.

### 4.1. Datasets

Experiments are conducted on nine commonly used multiview datasets, and the general information of the dataset is shown in Table 1.

- Caltech101 [59]: Coltech101-07 and Coltech101-20 are selected from the Caltech101 dataset, which includes 2386 and 1474 images, respectively. Each image contains six feature vectors of GABOR, WM (wavelet moment), CENT (Centrist features), HOG, GIST and LBP.
- NUS [60]: It contains 2400 images in 12 categories. The six features of colour histogram, CM, edge direction histogram, wavelet texture, block-wise colour moment and SIFT description are included for each image.
- ORL [61]: This dataset contains 400 images with four feature vectors of GIST, HOG, LBP, and CENT.
- 3sources: This dataset contains 169 news documents reported by three online news organizations, BBC, The Guardian and Reuters.
- BBC [62]: It is a collection of 685 documents from the BBC News website, each divided into four feature vectors.
- BBC_Sport [62]: This dataset consists of 544 documents collected from the BBC Sports website; each document has two feature vectors.
- 100leaves [63]: It consists of 1600 samples from the UCI repository, each of which is one of a hundred species.
- Scene15 [64]: It consists of 4485 images of indoor and outdoor scenes with a total of three views.

### 4.2. Evaluation Metrics

This paper adopts six evaluation indicators to measure the quality of clustering results, namely accuracy (ACC), Adjusted Rand Index (ARI), Normalized Mutual Information (NMI), F1-score (F), Purity and Precision.

(1). Accuracy: ACC refers to the ratio of the number of correctly clustered samples to the total number of instances N.

(12) $$Acc=\frac{TP+TN}{N}$$

where N=TP+FP+FN+TN, TP represents true positive, FP means false positive, FN indicates false negative and TN denotes true negative.

(2). Adjusted Rand Index: The value range of the ARI is [−1, 1].
  (13) $$\;\begin{array}{c} RI=\frac{TP+TN}{TP+FP+TN+FN}\\ ARI=\frac{RI-E\left[RI\right]}{\max(RI)-E\left[RI\right]} \end{array}$$
(3). Normalized Mutual Information: NMI measures the difference between cluster partitions through information theory. The value range is [0, 1]

(14) $$NMI\left(X;Y\right)=2\frac{I\left(X;Y\right)}{H\left(X\right)+H\left(Y\right)}$$

where I(X;Y) denotes the mutual information between random variables X and Y, and H(X), H(Y) are in the entropy of them.

(4). Precision: It represents the probability of the true positive sample among all predicted positive samples.
  (15) $$\;\;Precision=\frac{TP}{TP+FP}$$
(5). F1-score: F is the harmonic mean of precision and recall to comprehensively measure the clustering effect.
  (16) $$\begin{array}{c} R=\frac{TP}{TP+FN}\\ F=\frac{2*Precision*R}{Precision+R} \end{array}$$
(6). Purity: The general idea of cluster purity is to divide the number of correctly clustered instances by the total number of instances.

(17) $$\;Purity=\left(\Omega ,T\right)=\frac{1}{N}\underset{c}{\sum }\underset{j}{\max}\left|\omega_{c}\cap t_{j}\right|$$

where $\Omega =\left\{\omega_{1},\omega_{2},\ldots \omega_{c}\right\}$ denotes the clustered clusters, and $T=\left\{t_{1},t_{2},\ldots ,t_{j}\right\}$ represents the correct category. $\omega_{c}$ is all samples in the cth class after clustering. $t_{j}$ expresses the true positive sample in the jth cluster. Its value range is [0, 1]—the higher the better.

### 4.3. Compared Methods

To verify that the proposed method can effectively improve the clustering performance, we compare the MVCS-CP method with a single-view clustering method (spectral clustering method) and six state-of-the-art multiview clustering methods.

- SC [65] performs clustering on every single view and concatenates all views in the dataset into one view (Featconcat) for clustering.
- GBS [33] proposes a general graph-based multiview clustering system. The number of neighbors takes its default setting of 5.
- AMGL [66] is a parameter-free model for spectral embedding learning that automatically learns the weights for each view by solving a square root trace minimization problem.
- MVGL [67] uses it to explore the Laplacian rank-constrained graph after obtaining the similarity graph for each view, where the number of neighbors is set to a default value of 10.
- ASMV [32] adaptively jointly optimizes the data correlation between multiple features, and the number of neighbors is set to 15.
- CDMGC [36] is a graph clustering method of explicitly exploiting both multiview consistency and multiview diversity. The parameters in the experiment leverage the default values in the code provided by the author.
- CoMSC [68] is a multiview subspace clustering algorithm that groups objects and simultaneously removes data redundancy. In the experiment, the two parameters λ and c are respectively searched in $\{2^{-10},2^{-8},2^{-6},2^{-4},2^{-2},2^{0},$ $2^{2},2^{4},2^{6},2^{8},2^{10}\}$ and {k, 2k,…,20k}, where k is the number of classes.

The experimental results on the nine datasets are shown in Table 2, Table 3, Table 4, Table 5, Table 6, Table 7, Table 8, Table 9 and Table 10, with the standard deviation in parentheses. The value with the best experimental result is bolded, and the second-best value is marked with an underscore (_). The following results can be obtained from the tables:

- The proposed MVCS-CP method performs better on six evaluation metrics on all datasets, basically being the best or second best. In the caltech101-20 dataset, it has the best performance on the four metrics of ARI, NMI, precision and purity with 8%, 2%, 4% and 6% improvement over the second-best results. As far as the caltech101-7 dataset is concerned, the three indicators of ACC, Precision and Purity are the best, and the remaining indicators ARI, NMI and F are the second best. In terms of the NUS dataset, except for the F indicator, the rest of the indicators perform the best. Compared with the better overall performance of CoMSC, the effect was increased by 5% (ACC), 2% (ARI), 2% (NMI), 2% (Precision) and 16% (Purity), respectively. Synthesizing the caltech101-20, caltech101-7 and NUS datasets, it can be concluded that the proposed MVCS-CP can achieve better results in processing more than five views. MVCS-CP performs optimally on all six metrics for the ORL dataset, with an average of 2% improvement for each metric over the second-best result. As for the 100leaves dataset, except for the NMI indicator, which is 0.5% lower than the second-best, all other indicators perform the best. The ORL and 100 leaves datasets have more clusters numbers (40 and 100 categories, respectively). Based on the above experimental results, it can be found that MVCS-CP can cope well with rich clusters number. On the 3sources dataset and the BBC dataset, the proposed algorithm demonstrates obvious improvement on all indicators. In addition, the BBC_Sport dataset has the best performance on the remaining five metrics except for Precision, which is the second-best. Combining the three datasets of 3sources, BBC and BBC_Sport, all of them have a higher dimension of the order of thousands. It can be seen that the proposed MVCS-CP can achieve satisfactory results when dealing with datasets with higher dimensions.
- Furthermore, the Scene dataset has the best performance on four metrics (ACC, ARI, Precision and Purity), especially on Purity, which is 7% better than the second-best result. And it is comparable to the best results on the NMI indicator. This illustrates that MVCS-CP can handle larger-scale datasets.
- Compared with the state-of-the-art multiview clustering algorithms, the MVCS-CP algorithm has better or comparable performance. This suggests that taking each view’s geometry and sparse representation into account yields better results.
- In terms of the single-view method, it is found that the multiview clustering algorithm is basically better than it, which shows that considering the multiple features of the dataset can be better clustered. However, on the BBC_Sport dataset, Featconcat performs the best in terms of Precision, which means that the multiview clustering method still needs further improvement.

**Figure 5:**
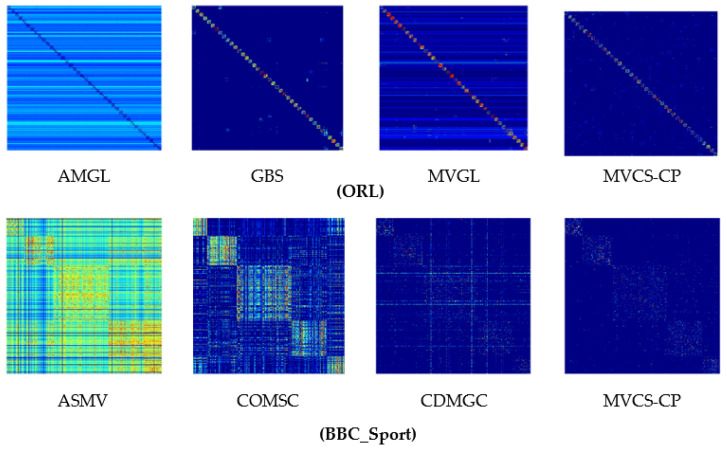
Unified graph of ORL and BBC_Sport.

In order to display the results more intuitively, the unified graph learned by different methods is visualized, taking ORL and BBC_Sport as examples to explain (see Figure 5). ORL dataset, the methods can obtain the correct number of block diagonals. Nevertheless, AMGL and MVGL have a lot of noise—the result of GBS is clearer, yet the noise is more than that of MVCS-CP. About the BBC_Sport dataset, ASMV and CDMGC cannot acquire the correct number of block diagonals, and the COMSC block diagonal structure is obvious but noisier. The visual display of the unified graph indicates that the sparse representation can effectively reduce the noise.

### 4.4. Running Time

**Figure 6:**
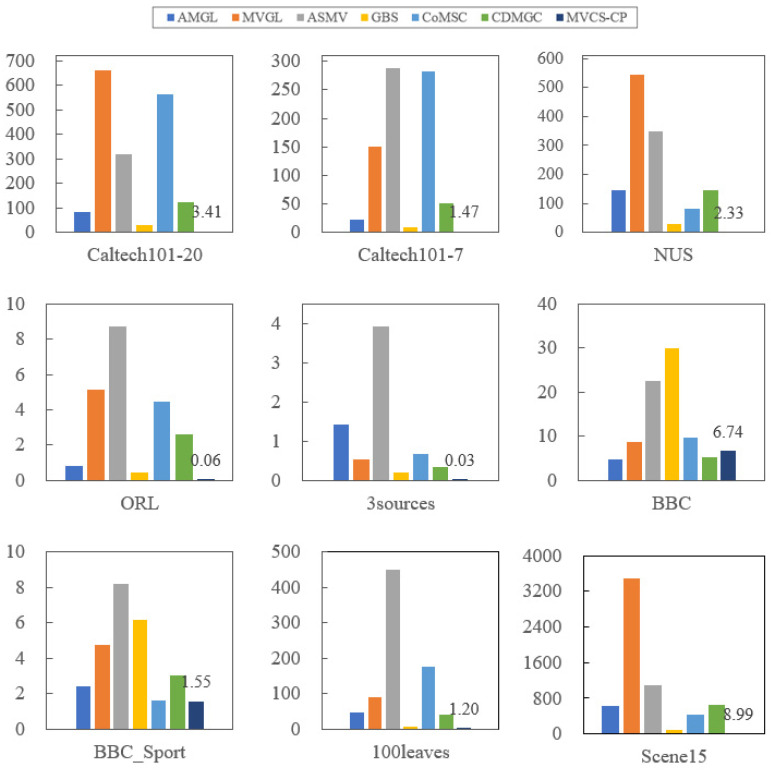
Performance comparison of running time on nine real-world datasets.

Table 11 (the value with the best experimental result is bolded) and Figure 6 show the runtime comparison of different multiview clustering methods on nine real-world datasets. It can be seen that except AMGL has the shortest running time on the BBC dataset; the proposed MVCS-CP method has the shortest running time on the remaining eight datasets. Even on a relatively large-scale Scene dataset, the time taken is less than 10 s. Compared with MVGL, ASMV and CoMSC methods, the MVCS-CP method has obvious advantages, and the time cost for most of the datasets is only one percent of the former methods. In conclusion, the method can save a significant amount of time without iteration.

### 4.5. Comparison of the Number of Neighbors

**Figure 7:**
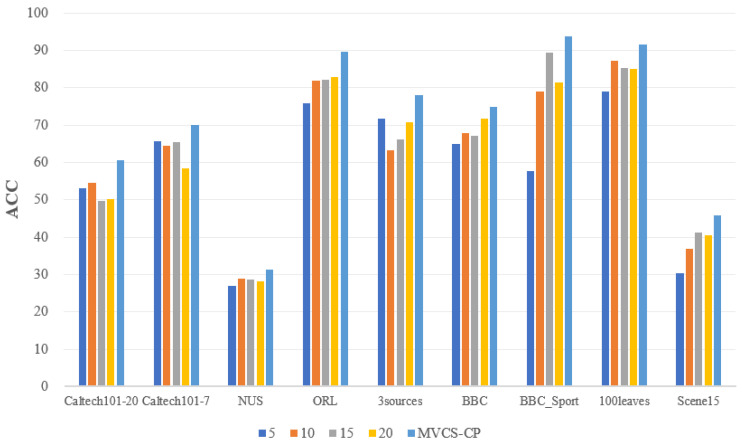
The comparison of ACC with fixed number and MVCS-CP.

**Figure 8:**
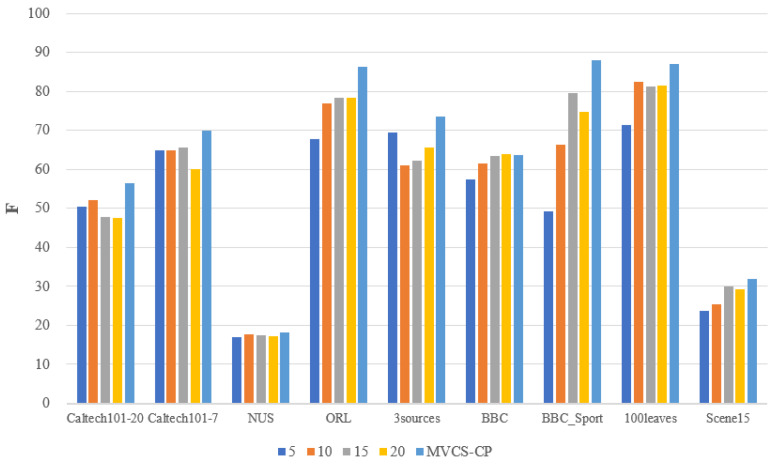
The comparison of F with fixed number and MVCS-CP.

Determining the number of neighbors in each view is an important step for the MVCS-CP method before constructing the similarity matrix, which is different from other methods. Table 12 indicates the different number of neighbors automatically determined for each view for the nine datasets. It can be seen that except the 3sources dataset has the same number of neighbors in each view, the rest of the datasets are different. In order to verify that automatically determining the number of neighbors can effectively improve the clustering results, the fixed number of MVCS-CP methods will be adopted for comparison. Figure 7 and Figure 8 show the comparison of the ACC and F metrics when the number of neighbors is fixed at 5, 10, 15, 20 and the number of neighbors is automatically determined, respectively. The MVCS-CP method has the best clustering effect on both ACC and F values, which show the effectiveness of automatically determining the number of neighbors.

### 4.6. Parameter Analysis

**Figure 9:**
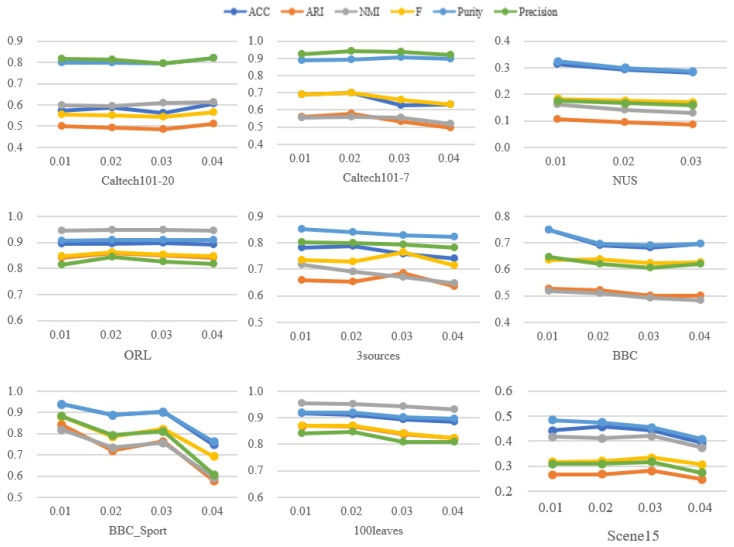
The influence of *para* changes on clustering effect indicators on nine datasets.

In this paper, the parameter para is used when forming the unified graph matrix, and its selection range is {0.01, 0.02, 003, 0.04}. It can be seen from Figure 9 that the clustering effect of MVCS-CP is relatively stable in the parameter range from 0.01 to 0.04. When the parameter of the NUS dataset is 0.04, the data is too complex to be read during clustering, so only the results from 0.01 to 0.03 are shown in the figure. Figure 9 demonstrates that the proposed method is less sensitive to parameters.

### 4.7. Result Discussion

As mentioned previously, the comprehensive results on nine common datasets indicate that the MVCS-CP can handle datasets with different numbers of views and clusters, different dimensions and different sizes. For higher-dimensional and larger-size datasets, it can still obtain better clustering results. The above shows that considering the geometry and sparse representation of each view enables better clustering. The visualization of the unified graph demonstrates that MVCS-CP obtains a clearer and more concentrated clustering structure. This shows that the introduction of sparse representation effectively reduces noise. In terms of the running time results, the MVCS-CP method without iterations saves more time. As far as the number of neighbors, automatically determining the number of neighbors can effectively improve the clustering results. Furthermore, MVCS-CP is not sensitive to parameters.

## 5. Conclusions

This paper proposes a multiview clustering of adaptive sparse representation based on coupled P system (MVCS-CP). After reading the data matrix, the number of neighbors of each view is automatically determined, and then it adopts the concepts of manifold learning and sparse representation to construct the similarity matrix. During the unified graph formation stage, it aims to learn a sparse similarity matrix that is as consistent as possible with all views. In addition, the model directly obtains the close-form solution without iteration, consuming less time. The experimental data on nine real datasets demonstrate that the proposed MVCS-CP method outperforms the state-of-the-art multiview clustering algorithms. Moreover, the comparison experiment with the fixed number of neighbors indicates that the automatic determination of the number of neighbors is effective. In brief, this method can be implemented quickly and intuitively, which is more suitable for dealing with practical problems. The method of embedding a deep neural network into multiview clustering to automatically determine the number of clusters and parameter-free will be issues worth exploring in the future.

## Figures and Tables

**Table 1:** The general information of the dataset (- means null).

Datasets	Objects	View	Clusters	*d*	*d*	*d*	*d*	*d*	*d*
Caltech101-20	2386	6	20	48	40	254	1984	512	928
Caltech101-7	1474	6	20	48	40	254	1984	512	928
NUS	2400	6	12	64	44	73	128	155	500
ORL	400	4	40	512	89	864	254	-	-
3sources	169	3	6	3560	3631	3068	-	-	-
BBC	685	4	5	4659	4633	4665	4684	-	-
BBC_Sport	544	2	5	3183	3203	-	-	-	-
100leaves	1600	3	100	64	64	64	-	-	-
Scene15	4485	3	15	20	59	40	-	-	-

**Table 2:** Experimental results on Caltech101-20 datasets (%).

Caltech101-20	ACC	ARI	NMI	Precision	F	Purity
SC1	26.55 ± 1.46	11.73 ± 1.03	26.99 ± 0.4	36.13 ± 1.79	19.47 ± 1.01	52.46 ± 0.92
SC2	28.32 ± 1.38	16.27 ± 0.26	33.43 ± 0.32	46.73 ± 0.86	22.96 ± 0.32	59.62 ± 0.63
SC3	28.32 ± 1.38	16.27 ± 0.26	33.43 ± 0.32	46.73 ± 0.86	22.96 ± 0.32	59.62 ± 0.63
SC4	40.49 ± 1.13	30.05 ± 1.67	52.89 ± 0.99	71.02 ± 1.78	35.78 ± 1.67	75.43 ± 0.68
SC5	39.28 ± 1.72	27.47 ± 1.85	48.82 ± 0.98	67.2 ± 2.4	33.31 ± 1.81	73.19 ± 1.15
SC6	35.44 ± 2.75	24.18 ± 1.93	43.31 ± 1.37	60.39 ± 3.51	30.39 ± 1.8	68.64 ± 1.6
Featconcat	49.97 ± 0.13	14.52 ± 0.35	20.2 ± 0.29	23.09 ± 0.19	36.51 ± 0.21	52.77 ± 0.12
AMGL	52.73 ± 3.14	26.82 ± 2.82	52.19 ± 3.33	35.21 ± 3.1	40.67 ± 1.99	67.62 ± 1.88
MVGL	60.69 ± 0	28.92 ± 0	50.73 ± 0	33.54 ± 0	44.15 ± 0	71.29 ± 0
ASMV	41.17 ± 2.07	28.79 ± 2.06	54.23 ± 0.65	63.13 ± 2.75	35.25 ± 2	74.78 ± 0.7
GBS	**64 ± 0**	34.08 ± 0	53.73 ± 0	37.07 ± 0	47.95 ± 0	73.34 ± 0
CoMSC	53.98 ± 4.83	43.01 ± 6.31	59.47 ± 6.59	78.6 ± 4.91	**78.21 ± 5.05**	48.77 ± 2.32
CDMGC	55.7 ± 9.49	22.72 ± 10.33	44.68 ± 8.29	29.28 ± 6.34	40.43 ± 6.76	65.08 ± 8.99
MVCS-CP	60.6 ± 0.59	**51.05 ± 2.2**	**61.36 ± 1.48**	**82.23 ± 1.56**	56.56 ± 2.07	**81.98 ± 1.01**

The value with the best experimental result is bolded, and the second-best value is marked with an underscore (_).

**Table 3:** Experimental results on Caltech101-07 datasets (%).

Caltech101-7	ACC	ARI	NMI	Precision	F	Purity
SC1	28.83 ± 2.1	7.94 ± 0.92	11.51 ± 0.5	48.77 ± 0.97	29.14 ± 1.45	65.88 ± 1.66
SC2	34.79 ± 2.24	19.67 ± 1.26	24.18 ± 0.59	66.23 ± 1.46	36.94 ± 1.13	73.09 ± 0.75
SC3	55.6 ± 0.22	2.81 ± 0.26	3.15 ± 0.37	39.44 ± 0.09	55.91 ± 0.03	56.61 ± 0.29
SC4	42.43 ± 2.57	29.55 ± 1.99	37.88 ± 1.62	78.48 ± 2.15	45.18 ± 1.9	81.25 ± 1.46
SC5	40.72 ± 0.39	28.11 ± 1.48	35.36 ± 0.7	77.99 ± 1.45	43.59 ± 1.37	81.41 ± 0.54
SC6	46.15 ± 3.24	30.32 ± 1.91	36.04 ± 1.17	78.42 ± 2.02	46.1 ± 1.64	80.62 ± 1.07
Featconcat	54.04 ± 0.04	1.22 ± 0.08	1.47 ± 0.03	38.93 ± 0.03	55.69 ± 0.06	54.52 ± 0.06
AMGL	64.46 ± 6.14	44.36 ± 5.82	54.6 ± 1.96	70.94 ± 6.65	63.71 ± 4.75	84.79 ± 0.77
MVGL	57.06 ± 0	45.96 ± 0	53.17 ± 0	87.25 ± 0	60.37 ± 0	87.04 ± 0
ASMV	40.77 ± 1.2	29.04 ± 1.22	41.55 ± 0.81	76.53 ± 0.75	45.2 ± 1.22	82.5 ± 0.53
GBS	69.2 ± 0	**59.43 ± 0**	**60.56 ± 0**	88.58 ± 0	**72.17 ± 0**	88.47 ± 0
CoMSC	63.28 ± 3.68	49.02 ± 3.96	53.62 ± 3.9	86.26 ± 4.95	63.49 ± 3.55	86.57 ± 1.32
CDMGC	51.74 ± 11.66	5.97 ± 23.25	23.71 ± 16	42.53 ± 12.76	50.26 ± 10.59	61.8 ± 12.3
MVCS-CP	**69.95 ± 0.03**	57.69 ± 0.07	56.13 ± 0.3	**94.27 ± 1.29**	69.99 ± 0.16	**89.48 ± 0**

The value with the best experimental result is bolded, and the second-best value is marked with an underscore (_).

**Table 4:** Experimental results on NUS datasets (%).

NUS	ACC	ARI	NMI	Precision	F	Purity
SC1	21.25 ± 0.42	4.32 ± 0.35	8.74 ± 0.19	12.03 ± 0.37	12.71 ± 0.22	22.99 ± 0.58
SC2	20.76 ± 0.42	4.23 ± 0.18	8.75 ± 0.34	12.04 ± 0.2	12.42 ± 0.09	22.41 ± 0.34
SC3	18.7 ± 0.22	3.4 ± 0.17	7.18 ± 0.23	11.33 ± 0.17	11.62 ± 0.15	19.94 ± 0.2
SC4	23.43 ± 1.1	5.23 ± 0.36	10.02 ± 0.61	13.02 ± 0.31	13.21 ± 0.36	24.84 ± 0.84
SC5	21.03 ± 0.45	4.73 ± 0.22	9.64 ± 0.72	12.44 ± 0.2	12.98 ± 0.22	22.41 ± 0.65
SC6	11.43 ± 0.18	0.32 ± 0.01	4.61 ± 0.14	8.44 ± 0.01	15.31 ± 0.02	13.09 ± 0.2
Featconcat	10.79 ± 0.23	0.32 ± 0.02	4.5 ± 0.16	8.44 ± 0.01	15.4 ± 0.02	12.75 ± 0.12
AMGL	21.43 ± 0.96	4.15 ± 0.66	12.2 ± 0.96	10.68 ± 0.48	16.33 ± 0.2	23.37 ± 0.99
MVGL	13 ± 0	0.36 ± 0	5.57 ± 0	8.46 ± 0	15.44 ± 0	13.83 ± 0
ASMV	12.13 ± 1.2	0.71 ± 0.84	8.13 ± 2.21	9.14 ± 0.94	14.21 ± 0.15	22.46 ± 2.67
GBS	16.5 ± 0	1.24 ± 0	7.88 ± 0	8.88 ± 0	15.92 ± 0	17.88 ± 0
CoMSC	26.83 ± 2.65	8.32 ± 3.47	14.12 ± 3.47	15.84 ± 2.98	**27.46 ± 2.76**	16 ± 1.49
CDMGC	11.96 ± 1.43	0.27 ± 0.25	4.14 ± 1.57	8.42 ± 0.12	15.42 ± 0.17	12.68 ± 1.54
MVCS-CP	**31.38 ± 0.83**	**10.49 ± 0.58**	**16.1 ± 0.29**	**17.52 ± 0.52**	18.21 ± 0.52	**32.42 ± 0.38**

The value with the best experimental result is bolded, and the second-best value is marked with an underscore (_).

**Table 5:** Experiments results on ORL datasets (%).

ORL	ACC	ARI	NMI	Precision	F	Purity
SC1	75.7 ± 1.95	68.01 ± 1.65	89.92 ± 0.68	58.42 ± 1.75	68.86 ± 1.6	80.6 ± 1.31
SC2	49.25 ± 2.02	35.32 ± 2.11	70.37 ± 1.16	34.84 ± 1.7	36.86 ± 2.07	53.3 ± 1.87
SC3	65.45 ± 1.16	58.83 ± 2.31	85.05 ± 0.81	51.09 ± 2.7	59.92 ± 2.23	71.8 ± 0.87
SC4	53.65 ± 2.06	37.44 ± 2.82	72.1 ± 1.5	36.77 ± 2.74	38.94 ± 2.76	57.15 ± 1.71
Featconcat	74.4 ± 0.72	68.87 ± 1.18	89.37 ± 0.41	60.55 ± 1.72	69.67 ± 1.14	79.5 ± 0.71
AMGL	72.91 ± 3.33	65.43 ± 6.51	89.69 ± 1.77	54.66 ± 7.71	66.39 ± 6.27	80.21 ± 2.54
MVGL	73.75 ± 0	52.74 ± 0	87.15 ± 0	40.38 ± 0	54.17 ± 0	80.25 ± 0
ASMV	67 ± 1.23	49.46 ± 0.67	81.08 ± 0.45	43.59 ± 1.37	50.79 ± 0.82	72.34 ± 0.71
GBS	83.75 ± 0	76.32 ± 0	92.6 ± 0	68.75 ± 0	76.92 ± 0	86.75 ± 0
CoMSC	86.5 ± 9.67	83.63 ± 13.03	94.42 ± 6.76	80.84 ± 11.97	84.01 ± 12.72	88.75 ± 9.78
CDMGC	71.35 ± 1.9	47.16 ± 3.27	86.7 ± 0.85	33.95 ± 3.15	48.88 ± 3.12	79.2 ± 0.96
MVCS-CP	**89.5 ± 2.71**	**85.96 ± 0.46**	**94.87 ± 0.08**	**84.27 ± 0.82**	**86.28 ± 0.45**	**90.75 ± 1.41**

The value with the best experimental result is bolded, and the second-best value is marked with an underscore (_).

**Table 6:** Experiments results on 3sources datasets (%).

3sources	ACC	ARI	NMI	Precision	F	Purity
SC1	30.3 ± 0.77	−2.87 ± 0.42	6.34 ± 0.73	22.06 ± 0.19	34.37 ± 0.53	36.45 ± 0.9
SC2	37.4 ± 0.77	4.58 ± 0.44	10.37 ± 1.6	25.19 ± 0.2	38.27 ± 0.2	39.76 ± 1.28
SC3	31.95 ± 0	−2 ± 0.19	7.07 ± 0.62	22.42 ± 0.08	35 ± 0.23	37.63 ± 0.79
Featconcat	31.01 ± 1.36	−0.37 ± 1.36	5.45 ± 2.12	23.09 ± 0.77	27.54 ± 1.84	37.28 ± 1.82
AMGL	34.02 ± 2.69	−1.66 ± 1.45	7.2 ± 2.95	22.58 ± 0.6	34.78 ± 0.56	39.25 ± 2.73
MVGL	30.77 ± 0	−3.38 ± 0	6.6 ± 0	21.86 ± 0	34.17 ± 0	37.87 ± 0
ASMV	69.82 ± 4.7	60.01 ± 7.14	64.07 ± 4.56	65.99 ± 6.45	69.84 ± 5.23	77.51 ± 3.75
GBS	69.23 ± 0	44.31 ± 0	54.8 ± 0	48.44 ± 0	60.47 ± 0	74.56 ± 0
CoMSC	64.93 ± 4.39	53.44 ± 5.59	62.41 ± 3.63	68.11 ± 4.98	63.54 ± 4.4	78.27 ± 3.18
CDMGC	34.91 ± 0	−1.26 ± 0.05	6.31 ± 0.26	22.73 ± 0.02	35.77 ± 0.08	39.35 ± 0.31
MVCS-CP	**78.11 ± 0.74**	**65.86 ± 1.27**	**71.62 ± 1.41**	**80.31 ± 3.49**	**73.49 ± 0.69**	**85.21 ± 0.56**

The value with the best experimental result is bolded, and the second-best value is marked with an underscore (_).

**Table 7:** Experiments results on BBC datasets (%).

BBC	ACC	ARI	NMI	Precision	F	Purity
SC1	33.11 ± 2.04	−1.4 ± 0.71	7.73 ± 2.46	22.88 ± 0.29	35.09 ± 0.39	36.15 ± 3.35
SC2	31.53 ± 0	−0.66 ± 0	1.24 ± 0.13	23.2 ± 0	37.26 ± 0	33.02 ± 0.07
SC3	30.92 ± 1.38	−0.71 ± 0.37	2.1 ± 0.16	23.17 ± 0.15	36.84 ± 0.6	33.28 ± 0.21
SC4	33.75 ± 0.28	−0.29 ± 0.13	2.71 ± 0.32	23.34 ± 0.05	37.24 ± 0.13	35.07 ± 0.52
Featconcat	33.26 ± 0.12	−0.23 ± 0.03	1.19 ± 0.07	23.37 ± 0.01	37.59 ± 0.03	34.01 ± 0.18
AMGL	35.66 ± 2.75	0.88 ± 1.22	2.23 ± 1.28	23.83 ± 0.51	37.22 ± 0.45	36.66 ± 2.93
MVGL	35.04 ± 0	0.24 ± 0	3.82 ± 0	23.55 ± 0	37.49 ± 0	36.35 ± 0
ASMV	63.94 ± 1.2	46.07 ± 3.02	46.82 ± 1.25	50.86 ± 0.86	0 ± 3.33	64.09 ± 1.21
GBS	69.34 ± 0	47.89 ± 0	48.52 ± 0	50.12 ± 0	63.33 ± 0	69.34 ± 0
CoMSC	70.18 ± 5.63	45.72 ± 8.07	51.49 ± 6.53	60.36 ± 6.92	57.99 ± 6.06	71.77 ± 3.89
CDMGC	31.53 ± 1.24	−0.69 ± 0.09	1.08 ± 1.03	23.19 ± 0.03	36.93 ± 0.13	32.99 ± 1.16
MVCS-CP	**74.89 ± 0.15**	**52.64 ± 0.19**	**51.76 ± 0.28**	**64.67 ± 0.11**	**63.56 ± 0.16**	**74.89 ± 0.15**

The value with the best experimental result is bolded, and the second-best value is marked with an underscore (_).

**Table 8:** Experiments results on BBC_Sport datasets (%).

BBC_Sport	ACC	ARI	NMI	Precision	F	Purity
SC1	35.59 ± 0.1	−0.07 ± 0.06	1.33 ± 0.05	23.83 ± 0.02	38.25 ± 0.04	36.54 ± 0.08
SC2	36.76 ± 0	0.36 ± 0.02	1.78 ± 0.06	23.99 ± 0.01	38.41 ± 0	37.1 ± 0.08
Featconcat	0.12 ± 0.12	1.4 ± 0.22	38.27 ± 0.08	**96.04 ± 0.38**	36.84 ± 0.28	23.9 ± 0.05
AMGL	36.21 ± 0	0.15 ± 0	1.34 ± 0.3	23.91 ± 0	38.42 ± 0.04	36.58 ± 0
MVGL	39.15 ± 0	1.89 ± 0	6.98 ± 0	24.59 ± 0	39.07 ± 0	39.52 ± 0
ASMV	69.12 ± 6.7	40.78 ± 5.49	39.26 ± 5.09	48.07 ± 4.8	57.76 ± 3.04	69.3 ± 5.95
GBS	80.7 ± 0	72.18 ± 0	72.26 ± 0	72.71 ± 0	79.43 ± 0	84.38 ± 0
CoMSC	88.6 ± 0.81	72.37 ± 2.66	71.63 ± 1.84	80.28 ± 0.99	78.84 ± 2.15	88.6 ± 0.81
CDMGC	36.03 ± 0.19	0.06 ± 0.14	1.43 ± 0.06	23.88 ± 0.05	38.33 ± 0.09	36.76 ± 0.19
MVCS-CP	**93.75 ± 0.41**	**84.18 ± 0.65**	**81.77 ± 0.44**	88.2 ± 1.88	**87.94 ± 0.78**	**93.75 ± 0.63**

The value with the best experimental result is bolded, and the second-best value is marked with an underscore (_).

**Table 9:** Experiments results on 100leaves datasets (%).

100leaves	ACC	ARI	NMI	Precision	F	Purity
SC1	41.78 ± 1.23	28.47 ± 1.17	67.71 ± 0.43	26.94 ± 1.16	29.2 ± 1.16	44.39 ± 1.29
SC2	33.3 ± 1.04	20.85 ± 0.84	62.44 ± 0.76	18.51 ± 0.76	21.72 ± 0.83	36.28 ± 1
SC3	45.96 ± 2.08	31.41 ± 1.85	70.13 ± 0.87	29.73 ± 1.91	32.1 ± 1.83	48.85 ± 1.86
Featconcat	62.91 ± 2.45	52.85 ± 2.42	82.01 ± 1.03	49.96 ± 2.59	53.32 ± 2.39	66.23 ± 2.15
AMGL	77.58 ± 2.5	47.47 ± 11.8	87.87 ± 2.17	34.87 ± 11.62	48.18 ± 11.58	81.25 ± 1.94
MVGL	81.06 ± 0	51.55 ± 0	89.12 ± 0	37.95 ± 0	52.17 ± 0	83.31 ± 0
ASMV	48.5 ± 0.41	23.8 ± 0.59	71.38 ± 0.51	16.36 ± 0.37	24.89 ± 0.19	54.06 ± 0.58
GBS	82.44 ± 0	57.11 ± 0	91.15 ± 0	42.67 ± 0	57.65 ± 0	85.13 ± 0
CoMSC	88.5 ± 6.83	86.56 ± 6.95	**95.95 ± 4.84**	82.92 ± 6.83	86.69 ± 6.2	90.88 ± 5.49
CDMGC	88.61 ± 1.34	76.15 ± 9.08	94.54 ± 1.1	66.56 ± 12.45	76.42 ± 8.95	89.93 ± 1.04
MVCS-CP	**91.5 ± 0.74**	**86.82 ± 0.17**	95.39 ± 0.13	**84.1 ± 0.43**	**86.95 ± 0.16**	**92 ± 0.32**

The value with the best experimental result is bolded, and the second-best value is marked with an underscore (_).

**Table 10:** Experiments results on Scene15 datasets (%).

Scene15	ACC	ARI	NMI	Precision	F	Purity
SC1	34.69 ± 0.7	19.64 ± 0.26	36.53 ± 0.19	24.87 ± 0.38	25.29 ± 0.23	40.08 ± 0.56
SC2	25.39 ± 0.43	10.05 ± 0.14	21.86 ± 0.29	14.06 ± 0.22	18.01 ± 0.1	27.87 ± 0.54
SC3	22.7 ± 0.99	8.82 ± 0.6	19.89 ± 0.18	14.77 ± 0.56	15.38 ± 0.57	28.54 ± 0.34
Featconcat	14.46 ± 0.59	1.58 ± 0.33	10.49 ± 1.05	7.68 ± 0.17	13.78 ± 0.16	17.33 ± 0.66
AMGL	32.78 ± 2.41	15.1 ± 1.73	30.79 ± 1.84	16.66 ± 1.6	23.38 ± 1.16	34.06 ± 2.09
MVGL	23.21 ± 0	6.01 ± 0	20.44 ± 0	10 ± 0	17.16 ± 0	24.41 ± 0
ASMV	34.09 ± 0.41	17.52 ± 0.48	33.74 ± 0.51	22.38 ± 0.54	23.51 ± 0.41	38.86 ± 0.69
GBS	14 ± 0	0.42 ± 0	5.82 ± 0	7.11 ± 0	13.17 ± 0	14.65 ± 0
CoMSC	43.15 ± 2.69	25.86 ± 1.97	**41.24 ± 1.39**	30.72 ± 2.02	47.29 ± 2.53	31.04 ± 1.79
CDMGC	12.44 ± 0.73	0.19 ± 0.13	3.99 ± 0.84	7 ± 0.06	13.01 ± 0.09	12.97 ± 0.71
MVCS-CP	**45.84 ± 2.12**	**26.71 ± 1.01**	41.18 ± 0.39	**30.85 ± 1.01**	31.95 ± 0.92	**47.42 ± 0.81**

The value with the best experimental result is bolded, and the second-best value is marked with an underscore (_).

**Table 11:** Performance comparison of running time on nine real-world datasets.

Time(s)	AMGL	MVGL	ASMV	GBS	CoMSC	CDMGC	MVCS-CP
Caltech101-20	80.954	662.011	317.870	28.231	562.93	122.846	**3.411**
Caltech101-7	21.3763	150.587	288.339	8.102	282.196	51.511	**1.467**
NUS	144.625	545.729	349.962	27.655	81.597	144.333	**2.333**
ORL	0.809	5.115	8.740	0.459	4.441	2.612	**0.062**
3sources	1.436	0.528	3.918	0.193	0.683	0.354	**0.028**
BBC	**4.837**	8.776	22.629	29.819	9.583	5.269	6.742
BBC_Sport	2.387	4.753	8.214	6.199	1.628	3.054	**1.552**
100leaves	47.818	90.341	449.571	5.849	175.703	40.080	**1.204**
Scence15	616.978	3485.092	1100.982	97.190	432.381	641.264	**8.988**

**Table 12:** The number of neighbors each view for nine datasets (- means null).

Datasets	*d*_1_	*d*_2_	*d*_3_	*d*_4_	*d*_5_	*d*_6_
Caltech101-20	18	18	21	33	25	33
Caltech101-7	17	16	21	28	31	27
NUS	13	2	4	5	5	16
ORL	7	10	8	19	-	-
3sources	8	8	8	-	-	-
BBC	16	13	9	15	-	-
BBC_Sport	14	16	-	-	-	-
100leaves	17	26	14	-	-	-
Scene15	24	18	31	-	-	-

## Data Availability

The datasets used in this paper come from related papers (see Section 4.1) or contact the authors for the full datasets.
